# Removal of peanut allergen Ara h 1 from common hospital surfaces, toys and books using standard cleaning methods

**DOI:** 10.1186/s13223-015-0069-x

**Published:** 2015-01-23

**Authors:** Wade TA Watson, AnnMarie Woodrow, Andrew W Stadnyk

**Affiliations:** Department of Pediatrics, Dalhousie University, Halifax, NS Canada; IWK Health Centre, 5850/5980 University Avenue, Halifax, NS Canada B3K 6R8

**Keywords:** Food allergy, Peanut allergen, Cleaning, Hospital, Contamination, Ara h 1

## Abstract

**Background:**

In children, a diagnosis of peanut allergy causes concern about accidental exposure because even small amounts of peanut protein could trigger an allergic reaction. Contamination of toys, books or other items by peanut butter in areas where individuals have eaten may occur in hospital waiting rooms and cafeterias. It is not known if hospital cleaning wipes are effective in removing peanut allergen.

**Objectives:**

The purpose of this study was to determine whether cleaning peanut contaminated items with common household and hospital cleaning wipes would remove peanut allergen.

**Methods:**

5 mL of peanut butter was evenly smeared on a 12 inch by 12 inch (30.5 by 30.5 cm) square on a nonporous (laminated plastic) table surface, a plastic doll, and a textured plastic ball, and 2.5 mL was applied to smooth and textured book covers. Samples for measurement of Ara h 1 were collected prior to the application of the peanut butter (baseline), and after cleaning with a common household wipe and two commercial hospital wipes. A monoclonal-based ELISA for arachis hypogaea allergen 1 (Ara h 1), range of detection 1.95-2000 ng/mL, was used to assess peanut allergen on each item. The samples were diluted 1:50 for testing.

**Results:**

At baseline, there was no detectable Ara h 1 allergen on any item at baseline. Detectable Ara h 1 was detected on all products after applying peanut butter (range 1.2-19.0 micrograms/mL).

After cleaning with any product, no Ara h 1 was detected on any item.

**Conclusions:**

Table surfaces, book covers and plastic toys can be cleaned to remove peanut allergen Ara h 1 using common household and hospital cleaning wipes. Regular cleaning of these products or cleaning prior to their use should be promoted to reduce the risk of accidental peanut exposure, especially in areas where they have been used by many children.

## Background

Peanut allergy affects approximately 1.6% of school age children [1]. Exposure to peanut, even in small quantities, is capable of causing life-threatening reactions [2,3]. Food allergy and specifically peanut allergy has a tremendous psychological burden on children and their families [4–10]. Having one food allergy impacts the introduction of other allergenic foods in allergic children and their siblings [11].

Families express major concern about accidental exposure to food in the child’s environment, leading to significant impact for them and the child. A survey of caregivers [12] reported that that food allergy impacted meal preparation activities in 60% of participants, family social activities in 50% and stress levels in 41%. Ten percent of caregivers did not send their child to school due to food allergy. School activities, such as field trips (59%) and school parties (68%), were significantly affected by food allergy. Sixteen percent of caregivers avoided going to restaurants, 11% avoided allowing their child to play at friends’ houses, 14% avoided daycare or aftercare, 10% to 11% avoided parties and sports, and 26% avoided camp and sleepovers because of the child’s food allergy.

Current treatment of peanut allergy includes avoidance of the trigger food and treatment of a severe reaction with epinephrine [13]. While future treatment may include oral induction of tolerance [14], nothing will replace avoidance measures as an effective preventative strategy.

A previous study [15] demonstrated that peanut allergen (Ara h 1) was not widely distributed in preschools and schools. Hand washing and cleaning table surfaces with common cleaning agents easily removed peanut allergen. A recent study investigated the distribution of peanut protein in the home environment using a polyclonal peanut ELISA. Peanut protein was completely removed from granite table tops after cleaning with detergent, but persisted after detergent cleaning of laminate and wooden table surfaces, pillows and sofa covers [16]. We have previously shown that peanut allergen persists on a laminate table surface for at least 110 days if no cleaning occurs, but cleaning of surfaces with a common household cleaner easily removed the allergen [17]. We recommended regular cleaning of table surfaces as a safety measure for all individuals with peanut allergy.

While avoidance measures and restricting peanut in common areas reduces risk, accidental contamination of toys or other items in areas where individuals have eaten may occur in homes with no allergic children, hospital waiting rooms and cafeterias. For hospital administrators the question of whether common hospital cleaners would effectively remove peanut allergen from various surfaces in the hospital often is asked.

The purpose of this study was to determine whether cleaning peanut contaminated items commonly found in a hospital waiting room with common household and hospital cleaning wipes would remove the peanut allergen Ara h 1.

## Methods

Samples for measurement of Ara h 1 were collected prior to the application of the peanut butter (baseline) by wiping a 37 mm glass fibre filter moistened phosphate buffered saline (PBS) containing 1% Tween 20 across each surface in the same manner. For a flat surface the filter was wiped in a “z” fashion across the surface. For a curved surface the filter was wiped in three concentric circles. 5 mL of peanut butter was smeared on a 12 inch by 12 inch square on a laminate table surface, a plastic doll, and a textured plastic ball. 2.5 mL of peanut butter was smeared on both smooth and textured book covers. The items were allowed to air dry for approximately five minutes. All items were then cleaned with a common household cleaning wipe and two different commercial hospital wipes. The item was wiped until there was no discernible peanut residue on the wipe. More than one wipe may have been used. Another sample for Ara h 1 was collected from the clean surfaces after the items air dried, in the same manner. Each experiment was performed once.

The household commercial cleaning cloth used was Clorox® Disininfecting Wipes (Clorox Company, Brampton, Ontario, Canada) Two hospital wipes were used: Ultrawipes™ (Wood Wyant Inc, Victoriaville, Quebec, CA) and Butcher’s PerCept RTU Wipes™ (Virox Technologies Inc., Oakville, Ontario, CA). The active ingredients are listed in Table 1.

**Table 1:** **Active ingredients of commercial cleaning wipes used**

**Product**	**Manufacturer**	**Ingredients (% w/v)**
Clorox® Disinfecting Wipes (household)	The Clorox Company of Canada Brampton, Ontario, CA	n-alkyl dimethyl benzyl ammonium chloride (0.1-0.2)
		n-alkyl dimethyl ethylbenzyl ammonium chloride (0.1-0.2)
		isopropyl alcohol (1–5)
Ultrawipes™ (hospital)	Wood Wyant Inc Victoriaville, Quebec, CA	didecyl dimethyl ammonium chloride (0.1)
Butcher’s PerCept RTU Wipes™ (hospital)	Virox Technologies Inc. Oakville, Ontario, CA	hydrogen peroxide (0.5)

A 37 mm glass fibre filter moistened phosphate buffered saline (PBS) containing 1% Tween 20 was used to sample all items. The filters were stored at −20 degrees Celsius until extraction. After thawing the filters but prior to the extraction, 1.5 ml of PBS-Tween 20 was added and the samples were left rotating overnight at 4 degrees Celsius. The following day the filters were squeezed to remove all the liquid to fresh tubes, and were tested for the peanut allergen, Ara h 1 by ELISA (INDOOR Biotechnologies, Charlottesville, Va) [18]. The samples were diluted 1:5 and 1:50 for testing and the protocol was conducted as provided by the manufacturer. The ELISA is based on the “sandwich” technique in which mouse monoclonal anti-Ara h 1 is coated onto plastic wells, the sample (or standard) applied, washed, then a second mouse monoclonal antibody added to detect the bound antigen. The concentration of antigen in the samples is interpolated from a standard curve derived from the relationship between purified antigen and absorbance determined by spectrophotometry. The standard curve is established at the same time and reagents as used on the samples. All samples were tested in triplicate. The range of detection of Ara h 1 was between 1.95 and 2000 ng/ml. After analysis the results were multiplied by the dilution factor and expressed as the actual concentration/mL (expressed as micrograms) for each sample.

## Results

At baseline, prior to peanut butter application, no detectable Ara h 1 was found on any item. Immediately post application, there was detectable Ara h 1 (range 1.2-19.0 micrograms/mL) on all items (Table 2). Immediately after cleaning with any wipe, no detectable Ara h 1 was found on any item.

**Table 2:** **Concentration of peanut allergen Ara h 1 on items at baseline, after applying peanut butter and after cleaning with each cleaning wipe**

**Item**	**Ara h 1 (micrograms/ml)**				
	**baseline**	**post-peanut**	**post-cleaning**		
			**Clorox®**	**Ultrawipes™**	**PerCept™**
Table	N.D.	3.5	N.D.	N.D.	N.D.
Ball	N.D.	1.2	N.D.	N.D.	N.D.
Smooth book cover	N.D.	19.0	N.D.	N.D.	N.D.
Textured book cover	N.D.	18.0	N.D.	N.D.	N.D.

N.D. = none detected.

## Discussion

It is reassuring that simple but thorough cleaning of toys, books and surfaces of many items that could be found in a hospital using common household or hospital cleaning wipes will remove the peanut allergen Ara h 1. Regular cleaning of these products or cleaning prior to their use should be promoted to reduce the risk of accidental peanut exposure, especially in areas where items have been used by many children. This information should be helpful to reduce concern in families of children with peanut allergy when they are in other homes with no allergic children, hospital waiting rooms and cafeterias, where one cannot guarantee that there has been no one recently consuming peanut butter.

There was variation with the amount of peanut protein found on the different surfaces, with the two books having the highest concentration. The likely explanation is that it was easier to apply and sample the books and there was a smaller area on which the peanut butter was applied. We wished to demonstrate the presence of Ara h 1 prior to the cleaning, and this was clearly present.

The threshold dose distribution of peanut has been measured in double blind, placebo-controlled studies in children. The protein dose at which 5% of the allergic population is likely to respond was reported at 1.6 mg for peanut [19]. The concentration of peanut protein on the surface of any of the products tested in our study was in the micrograms per mL, which is below the threshold of reaction based on this study. At the same time it is possible for cutaneous reactions to occur with contact on the skin.

Hospital cafeterias and waiting rooms could offer cleaning wipes to concerned families, which could definitely reduce risk and concern. Families could also carry household wipes with them, which could be used to clean toys or books before use.

There are several limitations in our study. We have not tested items which are more porous, for example wood, or cloth or loosely woven material in upholstered furniture. These items may be more difficult to clean as the peanut may penetrate the surface, which has been demonstrated by other investigators using detergent. In addition we did not test for the presence of Ara h 2 in our samples.

## Conclusions

Table surfaces, book covers and plastic toys commonly found in a hospital can be cleaned to remove peanut allergen Ara h 1 using common household and hospital cleaning wipes. Regular cleaning of these products or cleaning prior to their use should be promoted to reduce the risk of accidental peanut exposure, especially in areas where they have been used by many children.
